# Gut macrophage phenotype is dependent on the tumor microenvironment in colorectal cancer

**DOI:** 10.1038/cti.2016.21

**Published:** 2016-04-29

**Authors:** Samuel E Norton, Elliott T J Dunn, John L McCall, Fran Munro, Roslyn A Kemp

**Affiliations:** 1 Department of Microbiology and Immunology, University of Otago, Dunedin, New Zealand; 2 Department of Surgical Sciences, University of Otago, Dunedin, New Zealand

## Abstract

In contrast to many cancers, a high infiltration of macrophages in colorectal cancer (CRC) has been associated with improved prognosis for patients. Cytokines and other stimuli from the tumor microenvironment affect monocyte to macrophage maturation and subsequent phenotype and function. Heterogeneous myeloid populations were identified using a novel flow cytometry panel in both tumor and paired non-tumor bowel (NTB) from CRC patients. The frequency of macrophage subsets with a gut-conditioned phenotype was lower in tumor compared with NTB. We used an *in vitro* system to show that two of the macrophage populations represented pro-inflammatory and anti-inflammatory phenotypes. Conditioned media that contained high levels of interleukin-6 promoted and maintained an anti-inflammatory phenotype *in vitro*. This study demonstrates the plasticity and heterogeneity of macrophage subtypes in human CRC, and the feasibility of studying complex populations. *Ex vivo* experiments demonstrate that macrophage subsets are influenced by the tumor microenvironment.

Colorectal cancer (CRC) is the third most common cancer in men and the second in women worldwide. The highest estimated rates are in Australia and New Zealand (approximately 38.5 per 100 000 people).^1^ Immune cells have a critical role in both CRC progression and initiation, and infiltrating T cells have been associated with positive patient outcome.^2^ Furthermore, the immunoscore is a method of quantifying tumor-infiltrating T cells that has been proposed to improve prognostic power compared with clinicopathological disease stage.^3^

The role of tumor-associated macrophages (TAMs) in CRC remains inconclusive. TAMs are generally believed to have an anti-inflammatory, or M2-like, phenotype in cancer, supporting tumor growth and therefore are associated with poorer patient outcome.^4, 5^ However, unlike many cancers, a high infiltration of macrophages into colorectal tumors has been associated with improved patient prognosis.^6^ *In vitro* studies have highlighted a role for macrophages with a pro-inflammatory effect on CRC cell lines,^7, 8, 9^ although Kang *et al.*^10^ demonstrated that TAMs increased CRC cell invasiveness and migration. Importantly, these studies assessed the role of macrophages outside the gut, and it is likely that this microenvironment alters macrophage phenotype and function.

The plasticity of both *in vitro* derived and *ex vivo* macrophage populations was recently highlighted,^11^ leading us to hypothesize that the conflicting data surrounding the role of TAMs in CRC may be related to macrophage heterogeneity in the gut. This study had three aims: first, to develop an analysis strategy to allow the detection of multiple heterogeneous myeloid cell populations from human tissue; second, to use this strategy to differentiate populations of TAMs infiltrating colorectal tumors from macrophage populations present in the bowel; and third, to determine the effects of the tumor immune microenvironment on macrophage polarization. We have developed a novel flow cytometric analysis to identify macrophage phenotypes in CRC tumors and non-tumor bowel (NTB), acquired from patients undergoing surgery. Using this strategy, we identified four distinct macrophage populations in CRC tumors and found that these differ in frequency compared with paired NTB from the same patients. Furthermore, we found a lower frequency of tumor macrophages expressed markers associated with an anti-inflammatory phenotype compared with NTB macrophages. In order to postulate function from *ex vivo* macrophages, we cultured monocytes from peripheral blood mononuclear cells (PBMCs) and generated macrophage populations with analogous surface marker expression to those identified *ex vivo*. These populations had distinct pro- or anti-inflammatory functions. Finally, we showed that cytokines derived from the microenvironment changed the phenotype of *in situ* macrophages. Together, these data demonstrate the ability to reliably assess *in vivo* heterogeneity of TAMs in CRC and identify the tumor microenvironment as a potential target for improving antitumor immune responses.

## Results

### Identification of heterogeneous myeloid cell populations in colorectal tumors

**Figure 1:**
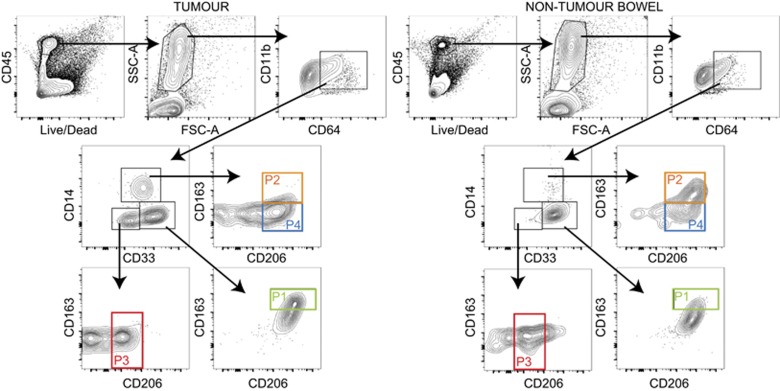
Macrophage populations in CRC are heterogeneous. Bowel tissue from patients with CRC was mechanically and enzymatically digested to extract single cells and surface expression was assessed by flow cytometry (left=tumor tissue, right=NTB tissue). Macrophages were identified as live CD45^+^SSC^hi^FSC^hi^CD64^+^CD11b^+^. Macrophages were assessed for CD14, CD33, CD206 and CD163 expression. P1 is CD14^−^CD33^+^CD163^+^; P2 is CD14^+^CD33^int^CD163^+^; P3 is CD14^−^CD33^−^CD206^lo^ and P4 is CD14^+^CD33^int^CD206^hi^. All gating determined by fluorescence minus one controls. Data shown are representative of 22 independent tissue samples.

Tissue macrophages were characterized by flow cytometry to identify specific populations in tumor and paired NTB from patients with CRC. Cells were gated for live CD45^+^ cells, followed by forward scatter (FSC) and side scatter (SSC),^12^ then assessed for expression of CD64, CD11b, CD33, CD14, CD206 and CD163. Macrophages were defined as live CD45^+^CD64^+^CD11b^+^ cells. CD64 significantly contributes to macrophage activation in response to IgG2A immunocomplex^13^ and is a recognized marker of human macrophages.^14^ CD11b is important in inducing activation in response to pathogen, or damage, associated molecular patterns.^15^ CD33 was used as a marker of gut macrophage conditioning^16^ and is associated with a suppressed phenotype in myeloid cells.^17^ CD14, CD206 and CD163 were used to define subset phenotypes. CD14 is the TLR4 co-receptor, CD206 is the macrophage mannose receptor and CD163 is a heme scavenger receptor—all of which are associated with macrophage function.^18, 19^ Our analysis revealed considerable patient to patient variation in surface marker expression; however, easily identified subsets were present in each tissue (Figure 1).

### Distinct myeloid phenotypes in the tumor: fewer macrophages expressed CD33^+^, CD64^hi^ or CD11b^hi^ in tumor compared with NTB

**Figure 2:**
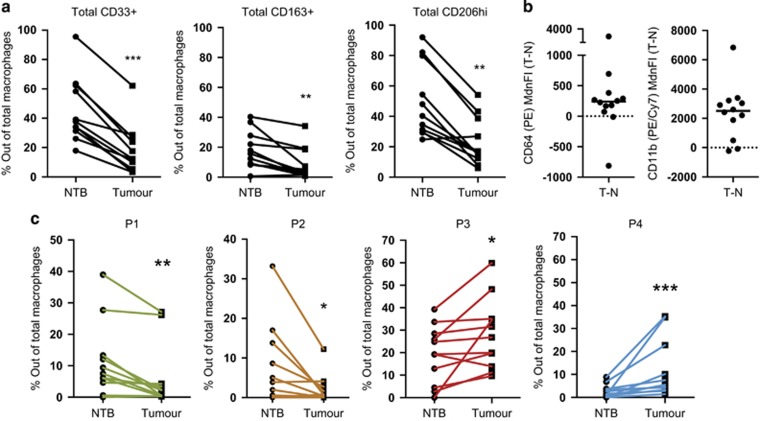
Fewer colorectal TAMs express CD33 but express increased CD11b and CD64 compared with NTB macrophages. NTB tissue and tumor tissue was removed by surgery from CRC patients. (**a**–**c**) Macrophages (CD45^+^SSC^hi^FSC^hi^CD64^+^CD11b^+^) were assessed for surface marker expression as in Figure 1. (**a**) Frequency of total macrophages expressing CD33, CD206^hi^ or CD163 in non-transformed bowel tissue (NTB) and tumor tissue (*n*=11, **P<0.01, ***P<0.001). (**b**) Median fluorescence intensity (MdnFI) difference (tumor minus NTB for each patient) for CD64-PE (left) and CD11b-PE-Cy7 (right) on macrophages. Dots above the broken line indicate higher expression on tumor macrophages than NTB macrophages for that patient. (**c**) Frequency of total macrophages with the P1–P4 phenotype in NTB and tumor tissue (*n*=11, *P<0.05, **P<0.01, ***P<0.001).

NTB was used in all experiments as a paired ‘non-tumor' gut environment control for tumor immune infiltrate. We were therefore able to assess changes to tumor-specific macrophage populations. First, we determined whether macrophage populations were present in different frequencies in tumor compared with NTB. We assessed changes in the frequency of cells from within the CD45^+^CD64^+^CD11b^+^ macrophage subset expressing CD14, CD163, CD206 or CD33. No significant difference was detected in CD14 expressing frequencies between tumor and NTB, possibly due to considerable variation within patients (data not shown). However, the frequencies of macrophages that were independently CD33^+^, CD163^+^ or CD206^hi^ were significantly lower in tumor compared with NTB (Figure 2a). Macrophage expression of CD64 and CD11b was higher in tumor compared with NTB (Figure 2b). These differences indicate a distinctive myeloid compartment within CRC tumors. A small number of patients with diverticulitis provided tissue samples. This control group showed variable frequencies of all macrophage populations studied and the patient number was not sufficiently high to establish a baseline frequency in non-cancer controls.

Gut-resident macrophages are CD33^+^ and CD14^−^.^20^ Furthermore, they have been characterized as lacking the most common macrophage markers including CD64 and CD11b.^16^ However, we found that CD33 expression was confined to CD45^+^CD64^+^CD11b^+^ macrophages in both tumor and NTB. In order to compare a spectrum of macrophage populations within the highly heterogeneous gut-resident population, we selected four populations present at high frequencies that were distinguishable based on positive surface marker expression of CD163 and CD206 in combination with other macrophage markers (P1–4; Figure 1). CD45^+^CD64^+^CD11b^+^ cells were divided into three populations based on CD33 and CD14 expression—CD33^−^CD14^−^ (P3), CD33^+^CD14^−^(P1) and CD33^int^CD14^+^ (P2 and P4). By using FSC^hi^SSC^hi^ gating to remove monocytes, we showed that gut macrophages can express CD64 and CD11b, contrary to previous reports.^20^ P1 was defined as CD33^+^CD14^−^. In most patients, CD33^+^CD14^−^ cells were the predominant macrophage population in NTB, but not in the tumor (Figure 1). P1 was also CD206^+^ and CD163^hi^. We observed a higher frequency of CD14^−^CD33^+^ cells in NTB compared with tumor, and a lower frequency of CD14^−^CD33^−^ cells in NTB compared with tumor. Although CD33^+^ is a characteristic marker of gut-resident macrophages, we were still confident that these cells were macrophages based on expression of CD64 and CD11b. P3 was CD33^−^ and CD14^−^; this population was observed predominantly in tumor tissue (Figure 1). P3 was also CD206^+/lo^ and CD163^−^. Subsets P2 and P4 were CD33^int^, CD14^+^ and CD206^+^. P2 was CD163^+^, whereas P4 was CD163^−^.

Both P1 and P2 populations were present at significantly lower frequencies in tumor compared with NTB, whereas the frequencies of P3 and P4 populations were higher in tumors compared with NTB (Figure 2c). These data demonstrate that the tumor microenvironment contains distinctly different macrophage subsets than paired NTB from the same patients.

As our approach measures changes in frequencies rather than total cell number, a change in frequency of one population may result in a corresponding change in frequency of a second population. We therefore studied the relationship between the frequencies of different populations. A decreased frequency of P1 cells correlated significantly with an increased frequency of P3 cells in the tumor, suggesting an inverse relationship between these two cell populations within the tumor microenvironment (Supplementary Figure 1). Further correlations were observed between each subset within either tissue. There were both positive and negative relationships between the four subsets (Supplementary Figure 1).

Taken together, these results suggest that macrophage populations within the tumor are distinct from those in NTB.

### Tissue macrophage populations have a similar phenotype to cultured pro-inflammatory and anti-inflammatory macrophages

**Figure 3:**
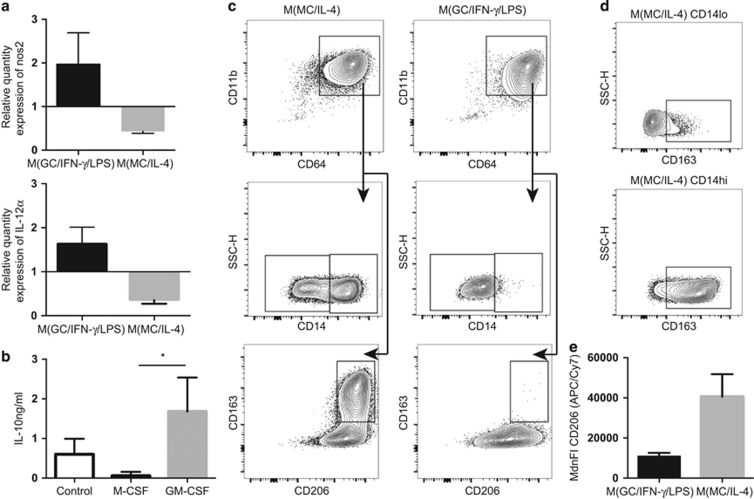
P3 and P4 cells represent tissue macrophage populations analogous to pro-inflammatory and anti-inflammatory macrophages. Macrophages from PBMCs of healthy people were cultured in GM-CSF+IFN-γ+LPS (M(GC/IFN-γ/LPS)) or M-CSF+IL-4 ((M(MC/IL-4)) and assessed by quantitative PCR (qPCR), ELISA and flow cytometry. All gating determined by fluorescence minus one controls. (**a**) M(GC/IFN-γ/LPS) and M(MC/IL-4) macrophages were assessed for expression of *nos2* and *IL-12a* by qPCR. Plots show relative expression compared with monocytes matured on plastic alone using ΔΔCT method and normalized to the housekeeping gene, GAPDH (*n*=3). (**b**) IL-10 production measured by ELISA, plots show supernatant from macrophages cultured with no supplement (control), GM-CSF or M-CSF. All cultures were stimulated with LPS for 2 h before supernatant extraction and analysis. (*n*=3, *P<0.05). (**c**) M(GC/IFN-γ/LPS) and (M(MC/IL-4) macrophages (liveFSC^hi^SSC^hi^CD64^+^CD11b^+^) were assessed by flow cytometry for expression of CD14, CD206 and CD163. Plots show macrophage identification by CD64 and CD11b followed by CD14 and CD206/CD163 expression for both cultures. Plots are representative of at least five experiments. (**d**) CD163 expression on CD14^−/lo^ or CD14^+/hi^ from M(MC/IL-4) macrophages gated as in **c**. (**e**) Median fluorescence intensity of CD206 (APC/Cy7) on liveFSC^hi^SSC^hi^CD64^+^CD11b^+^ M(GC/IFN-γ/LPS) and (M(MC/IL-4) macrophages (*n*=3).

Functional analysis of human primary macrophages is challenging, however, analysis of analogous populations *in vitro* can be indicative of macrophage function.^11^ PBMCs from healthy volunteers were cultured under controlled conditions to generate specific macrophage populations with cell surface phenotypes similar to those described *ex vivo*. Culture conditions were based on Mia *et al.*^21^: granuloycte macrophage colony-stimulating factor (GM-CSF), interferon (IFN)-γ and lipopolysaccharide (LPS) were added to promote a pro-inflammatory phenotype (M(GC/IFN-γ/LPS)), and macrophage colony-stimulating factor (M-CSF) and interleukin (IL)-4 to generate an anti-inflammatory phenotype (M(MC/IL-4)).^21^ These *in vitro* differentiated populations were then assessed by flow cytometry for co-expression of CD11b and CD64 as macrophage markers and CD14, CD206 and CD163 as previously described (Figures 1,3c–e). CD33 is not expressed on most mature tissue macrophage populations and so was not included in the characterization of these cells.^16^

Both M(GC/IFN-γ/LPS) and M(MC/IL-4) macrophages expressed CD64 and CD11b. M(GC/IFN-γ/LPS) were CD14^−/lo^, CD206^+/lo^ and CD163^−^. M(MC/IL-4) macrophages, however, were predominantly CD14^+/hi^, CD206^hi^ and CD163^+^ (Figure 3c). Interestingly, only CD14^hi^ M(MC/IL-4) macrophages were CD163^+^, which to our knowledge has not previously been described (Figure 3d). The remaining CD14^−/lo^ cells were CD163^−^ yet still retained higher expression of CD206 than M(GC/IFN-γ/LPS) macrophages (Figure 3e).

Monocytes cultured with GM-CSF, IFN-γ and LPS (M(GC/IFN-γ/LPS))^11^ had higher expression of *il-12a* and nitric oxide synthase (*nos*)*2* relative to glyceraldehyde-3-phosphate dehydrogenase (GAPDH) compared with non-stimulated controls, whereas monocytes cultured with M-CSF and IL-4 (M(MC/IL-4))^11^ had lower expression of *il-12a* and *nos2* (Figure 3a). When further stimulated with LPS, M(MC/IL-4) macrophages, but not M(GC/IFN-γ/LPS) macrophages, produced IL-10 (Figure 3b).

Thus, P3 macrophages described *ex vivo* expressed a surface marker profile analogous to cultured M(GC/IFN-γ/LPS) pro-inflammatory macrophages. P4 macrophages expressed a surface marker profile analogous to anti-inflammatory CD163^−^ M(MC/IL-4) macrophages. Although primary functional data would support these claims, analysis of *ex vivo* function of macrophages is difficult because of changes in surface phenotype during even short periods of *in vitro* culture.^11^

### IL-6 is associated with macrophage phenotype within the gut microenvironment

**Figure 4:**
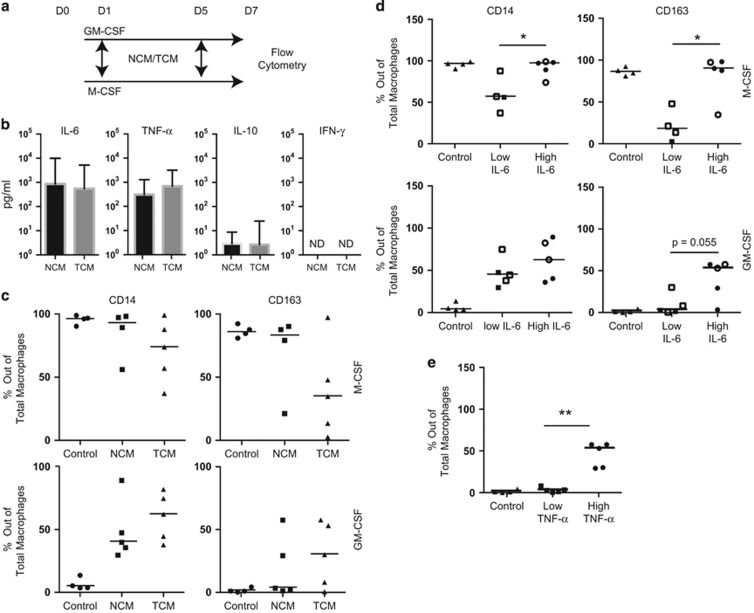
Colorectal tissue conditioned media with a high IL-6 concentration promotes anti-inflammatory surface marker expression. (**a**) PBMCs were isolated from patient blood at day 0. NCM or TCM was added to cells at either day 1 or day 5 before extraction on day 7. (**b**) Conditioned media was produced from patient tumor (TCM) and matched non-transformed bowel tissue (NCM). IL-6, TNF-α, IFN-γ and IL-10 concentration (pg ml^–1^) was assessed in each media by multiplex assay (*n*=4–5). (**c**–**e**) Macrophages were cultured with M-CSF or GM-CSF supplemented with conditioned media at day 1 of a 7-day culture. Plots show percentage macrophages expressing CD14 or CD163 out of the total macrophages (liveFSC^hi^SSC^hi^CD64^+^CD11b^+^). (**c**) Macrophages cultured in M-CSF (top) or GM-CSF (bottom) supplemented with nothing (control), TCM or NCM (*n*=4–5). (**d**) Conditioned media were stratified based on IL-6 concentration, regardless of tissue type, into IL-6lo (below the median) and IL-6hi (above the median). Plots show percentage macrophages expressing CD163 or CD14 from macrophage cultures with control (no conditioned media), IL-6lo or IL-6hi conditioned media added. Open symbol: TCM, solid symbol: NCM (*n*=4–5, *P<0.05). (**e**) Conditioned media were stratified based on TNF-α concentration, regardless of tissue type, into TNF-αlo (below the median) and TNF-αhi (above the median). Plots show percentage macrophages expressing CD163 from macrophage cultures with control (no conditioned media), IL-6lo or IL-6^hi^ conditioned media added (*n*=4–5, **P<0.01).

Macrophages are highly plastic cells and so their local microenvironment is essential in modulating their function.^22^ We showed that the macrophage population in colorectal tumors is different to that of paired NTB (Figure 2) and proposed that the tumor microenvironment may have a role in modulation of local macrophage populations. To investigate this hypothesis further, conditioned media was generated from both tumor (TCM) and NTB (NCM) to act as an *ex vivo* model of the specific microenvironment. Each conditioned media was analyzed for IL-6, tumor necrosis factor (TNF)-α, IL-10 and IFN-γ content using a multiplex assay. IFN-γ and IL-10 polarize macrophages toward a pro- or anti-inflammatory phenotype, respectively, and IL-6 and TNF-α have been associated with poor prognosis in many cancers.^23^ In addition, IL-6 has known effects on macrophage polarization,^24, 25^ and we were interested whether it would still have an effect within the tumor microenvironment. IFN-γ and IL-10 were not detectable in any conditioned media (Figure 4a). IL-6 and TNF-α were present at high concentrations in both TCM and NCM; however, inter-patient variation was high (IL-6: NCM=2567 pg ml^–1^±4153, TCM=1505 pg ml^–1^±2104, mean+s.d., *n*=4–5).

PBMCs were cultured with M-CSF/IL-4 or GM-CSF/IFNγ/LPS as for Figure 3. To assess the effect of the local environment, cultured media from tumors (TCM) or NTB (NCM) of matched patients was added to cultures at day 1 or day 5 of a 7-day culture (Figure 4a). Conditioned media was added at day 1 to assess its effect on monocyte maturation and at day 5 to examine its effect on modulating matured populations. Macrophages were then assessed by flow cytometry for expression of surface markers. There was no change in surface marker expression of M-CSF or GM-CSF macrophages cultured with NCM or TCM at day 5 (data not shown). However, monocytes cultured with either M-CSF or GM-CSF supplemented with NCM or TCM at day 1 had altered surface expression of CD14 and CD163 compared with monocytes cultured without conditioned media (Figure 4c). There was no significant difference in phenotype of cells cultured with TCM and NCM.

As the conditioned media contained variable amounts of cytokines from each patient, we stratified data from cultures of conditioned media from individual donors into high and low. This stratification was based on concentrations of IL-6 or TNF-α concentrations sitting above or below the median, and was performed regardless of tissue type, as no difference was seen between TCM and NCM (Figure 4b). M-CSF cultured monocytes supplemented with IL-6^hi^ conditioned media maintained high expression of CD14 and CD163, whereas those supplemented with IL-6^lo^ media showed significantly reduced expression of these two markers (Figure 4d). Macrophages cultured in IL-6^hi^ conditioned media in the presence of GM-CSF had higher expression of CD163 than macrophages cultured in IL-6^lo^ conditioned media, which maintained low expression of CD163. Similarly, CD163 expression was upregulated in GM-CSF cultured monocytes supplemented with TNF-α^hi^ conditioned media when compared with TNF-α^lo^ conditioned media (Figure 4e).

Taken together, these data indicate that a microenvironment with high IL-6 is associated with a macrophage phenotype expressing high CD14 and CD163, and identify IL-6 as a potential modulator of macrophage populations in the gut microenvironment.

## Discussion

In this study, we have shown the heterogeneity of macrophage populations in tumor and NTB from patients with CRC. We have also highlighted differences in the local microenvironment that may influence macrophage phenotype and function and, potentially, patient outcome.

An important goal of this work was to develop a useful framework for studying multiple populations of cells that could accurately reflect macrophage heterogeneity. Several groups have used flow cytometry or immunofluorescence/immunohistochemistry techniques to analyze human or mouse myeloid populations. However, given the heterogeneity and plasticity of macrophage populations, highlighted by Murray *et al.,*^11^ an analytical approach using *combinations of multiple markers* is essential to correctly identify sub-populations. This approach avoids oversimplifying experiments and prevents incorrect interpretation of the possible outcomes associated with the presence of a particular population. In support of this approach, we have published similar studies looking at T cells, and shown that multiple T-cell subsets with potentially very distinct functions, can co-exist in one site.^26, 27^ These populations are overlooked and undervalued if studied at a pan-T-cell marker level. To our knowledge, this is the first study to attempt to define multiple populations of macrophages from the human gut. Our analytical approach provides a framework for others to study these tissues in a variety of diseases and to understand the effect of multiple myeloid populations.

Separating populations P1–4 using multi-parametric flow cytometry is an effective approach to defining subsets within a spectrum of gut-conditioned macrophages. Previous reports have described gut-resident macrophages as CD33^+^ and CD14^−^,^16^ thus P1 represents this homeostatic population. These cells were observed at a higher frequency in NTB compared with tumor, which is consistent with this observation. P3 do not express CD33, CD14 or CD163, but express low levels of CD206, analogous to M(GC+IFN-γ+LPS) or M1-like macrophages cultured *in vitro*. The frequency of this subset was found to be higher in tumor compared with NTB. Furthermore, the macrophage activation markers, CD64 and CD11b,^13, 15^ were increased in expression intensity on TAMs compared with those found in NTB. Taken together with the lower frequency of CD33^+^ macrophages, and given that CD33 expression is associated with a suppressed myeloid phenotype,^17^ this suggests that CRC associated macrophages may be more activated than NTB macrophages.

The role of macrophages in the outcome of CRC remains unclear. Macrophages cultured *in vitro* in the presence of CRC cell lines displayed pro-inflammatory characteristics compared with M-CSF cultured macrophages.^8^ Our preliminary experiments revealed that CD68 was no better at detecting the macrophage population than the combination of CD11b and CD64 and so was not included in these experiments, especially given that CD64 is an accepted marker of human macrophages.^14^ However, CD68^+^ macrophages were associated with greater CD3^+^ cell infiltration in patient samples and produced IFN-γ^+^ (10–50% of total CD68^+^), suggesting these cells have a pro-inflammatory phenotype.^8^ Nagorsen *et al.*^6^ reported a trend toward improved survival associated with increased stromal CD163^+^ macrophages (CD68^+^) in CRC. However, as shown here, macrophages rarely exist in a homogenous population and there are limited studies analyzing heterogeneous macrophage populations in tumor models. Even in culture, macrophages display heterogeneity (Figure 3).

Mature macrophage populations are not terminally polarized. Macrophages can switch from pro- to anti-inflammatory phenotype within 24 h under the appropriate stimulus.^22^ The tumor microenvironment may promote the development of distinct macrophage populations from previously matured gut-resident phenotype macrophages. In support of this, we showed that the tumor microenvironment was associated with a higher frequency of P3 macrophages compared with NTB from the same patients. It is also possible that the tumor modulates homeostatic monocyte conditioning in the gut, promoting a more conventional phenotype. Media conditioned by tumor or NTB was linked to phenotypic alterations in monocyte–macrophage maturation (Figure 4). However, mature macrophage populations stimulated with the same conditioned media did not show the same phenotypic changes after 48 h (data not shown). Studies of macrophage differentiation in inflammatory bowel disease revealed that local inflammation led to disrupted monocyte maturation in the gut resulting in a dysregulated resident macrophage population.^28^ Taken together, this suggests that the tumor microenvironment could drive monocyte maturation toward a distinct macrophage phenotype, rather than modulating the mature macrophage population.

Patient serum levels of IL-6 have been associated with poor prognosis in many cancers, including CRC.^29^ IL-6 promotes cell survival and supports the production of vascular endothelial growth factor from both tumor and immune cells.^29^ Monocytes cultured in GM-CSF media with high IL-6, compared with no additional IL-6, had higher expression of CD163 and CD14. High expression of both markers is associated with an anti-inflammatory phenotype as seen in M(MC/IL-4) macrophages.^18^ Interestingly, IL-6^lo^ gut-conditioned media reduced expression of CD163 and CD14 in M-CSF cultures compared with both IL-6^hi^ conditioned media and conditioned media-free controls. We propose that macrophage differentiation is driven toward an anti-inflammatory, or wound healing, phenotype in an IL-6^hi^ gut environment. Therefore, as shown in other models,^24, 25^ IL-6 within the local tumor microenvironment of people with CRC, may modulate macrophage function.

The main limitation of this work is the lack of primary functional data. As previously stated, macrophages are highly plastic and thus *in vitro* experiments are often not accurate models of tumor–macrophage interactions. Attempts to measure *ex vivo* cytokine production or phagocytosis were confounded by changes in surface phenotype as a result of *in vitro* culture. Predicting macrophage function was improved by associating a combination of markers with a particular functional phenotype *in vitro*, as suggested by Murray *et al.*^11^ Furthermore, the phenotypes described here are in concordance with previously described populations. CD206 and CD163 are regularly used as markers of anti-inflammatory macrophage populations.^18^ We have initiated studies to correlate this phenotype with CD80/86 expression and assess the ability to activate T-cell responses.^30^

There was wide variation between patients, likely due to differences in tumor biology, but also because of the heterogeneity of CRC.^31^ Microsatellite instability status for our cohort is shown in Table 1, and is unlikely to be a cause of inter-patient variation.^32^ Future studies will attempt to associate specific immune populations with microsatellite instability status. Our previous data, and those from other groups, indicate no differences in the frequency of immune cell populations throughout the healthy gut; therefore, we do not believe that the tumor site will affect the frequency of infiltrating macrophage cells.^27, 33^ Despite the variation, the trend of increases in particular populations was consistent across all patients, indicating that macrophage populations are influenced more by the local microenvironment than underlying causes of disease.

Macrophages are highly represented in CRC immune infiltrates.^29^ TAMs are capable of inhibiting tumor progression and are thus associated with an improved patient prognosis, although evidence for the exact mechanism is inconclusive. Here we have shown that macrophage infiltrates into tumors are heterogeneous and distinct from NTB macrophage populations. This work highlights the importance of assessing macrophages at a subset level, appreciating that they do not exist as a uniform population with a single functionality. It also demonstrates the plasticity of intestinal macrophage populations particularly in the context of CRC and thus opens up strategies for their manipulation therapeutically.

## Methods

### Tissue collection

Tissue samples for both *ex vivo* and conditioned media analyses were obtained from patients undergoing elective surgery for CRC at Dunedin Hospital. The study was approved by the Lower Regional Ethics Committee (LRS/11/04/017/AM02) and all patients gave written informed consent before inclusion in the study in accordance with the Treaty of Helsinki. Resected specimens (one biopsy per patient site provided) were dissected fresh by a pathologist, and samples were obtained from the tumor (S1–S3)^26^ and adjacent NTB. The NTB samples were obtained from macroscopically normal appearing colon >10 cm from the tumor. Sixteen total patients were included in this study. Investigators were blinded to clinical data until after analysis was completed. Patient characteristics and microsatellite instability status for 11 samples in Figure 2 are shown in Table 1.

### Tissue processing

In the experiments described here, the total number of myeloid (CD45^+^CD64^+^ FSC^hi^ SSC^hi^ cells) was approximately 12 000 per mg of tumor tissue and 2 000 per mg of NTB tissue; average tissue weights are 140+100 mg for tumor and 460+215 mg for NTB tissue. These numbers are sufficient to provide meaningful data via flow cytometry.^34^ Data are expressed as frequencies rather than total cell number^26, 27, 34, 35, 36, 37^ as size of biopsies differed between patients. Samples were maintained in sterile phosphate-buffered saline (PBS; Invitrogen, Carlsbad, CA, USA) at 4 °C for no longer than 3 h until transportation where they were kept on ice. Samples were washed in sterile PBS and suspended in Roswell Park Memorial Institute medium (RPMI)-10 (Gibco, Green Island, NY, USA)—10% fetal calf serum (PAA Laboratories, Morningside, QLD, Australia)+100 μg ml^–1^ penicillin, 100 μg ml^–1^ streptomycin, 55 μM 2-mercaptoethanol (Sigma Aldrich, St Louis, MO, USA) with 0.5 mg ml^–1^ collagenase (Invitrogen) and incubated at 37 °C, 5% CO_2_ for 1 h. The tissue was then mechanically dissociated with a sterile scalpel. The tissue suspension was removed from the well and filtered with a 70 μm cell strainer (BD Falcon, Franklin Lakes, NJ, USA) into a 50 ml Falcon tube. The well was washed with PBS and filtered to remove any potentially adhered macrophages from the plastic.

### Flow cytometry

The following antibodies were used for both *ex vivo* and *in vitro* flow cytometry: CD45-BV605 (H130, 1 in 40, #304041), CD64-PE (10.1, 1 in 80, #305008), CD11b-PE-Cy7 (ICRF44, 1 in 160, #301322), CD14-BV421 (M5E2, 1 in 80, #301830), CD33-PerCP/Cy5.5 (WM53, 1 in 40, #303414), CD206-APC-Cy7 (15-2, 1 in 40, #321120) and CD163-APC (GHI/61, 1 in 80, #333610). All antibodies were titrated from 1 in 20 to 1 in 160. Dead cells were excluded using Live/dead fixable PE-Texas Red stain (Invitrogen). All flow cytometry antibodies were purchased from BioLegend (San Diego, CA, USA) unless otherwise stated, and the same antibodies were used to analyze cultured PBMCs, as well as *ex vivo* macrophage populations isolated from patients. Flow cytometric acquisition was performed on an LSR-FORTESSA using FacsDIVA (version 6.2, BD Biosciences, San Diego, CA, USA). Data were exported as FCS 3.0 files and analyzed using FlowJo (version X.0.7, Tree Star, Ashland OR, USA). All gates were set based on fluorescence minus one controls.^38^ Median fluorescence intensity was calculated using FlowJo software.

### *In vitro* macrophage culture

To generate monocyte derived-macrophages, PBMCs were isolated from blood using a Ficoll gradient (Ficoll-Paque plus, GE Healthcare, Piscata, NJ, USA) and cultured for 7 days in RPMI-10 and supplemented with 50 ng ml^–1^ M-CSF (Sigma Aldrich) or 20 ng ml^–1^ GM-CSF (BioLegend) in flat bottom polystyrene six-well plates (Corning, Corning, NY, USA). Plates were washed after 2 h post seeding to remove non-adherent cells. GM-CSF cultures were stimulated with IFN-γ (50 ng ml^–1^) (BD Biosciences) and LPS (10 ng ml) (Sigma Aldrich) and M-CSF cultures with IL-4 (20 ng ml) (R & D Systems, Minneapolis, MN, USA) on day 6. Enzyme-free dissociation buffer (Gibco; 30 min, 37 °C, 5% CO_2_) was used in conjunction with mechanical dissociation to remove cells from plastic after 7 days. Cells were then analyzed for gene expression as described below. Cell culture supernatants were analyzed for IL-10 production by enzyme-linked immunosorbent assay (ELISA) using standard protocols. Briefly, 96-well plates (BD Falcon, Microtest 96-well ELISA plate) were coated with purified anti-IL-10 capture antibody (BD Biosciences) and incubated at 37 °C for 1 h. Plates were then washed thoroughly (at least six times) in wash buffer (PBS+0.05% Tween 20). This washing procedure was completed between each step of the protocol. Plates were blocked with blocking buffer (PBS+1% bovine serum albumin) and incubated at 37 °C for 1 h. Supernatant samples and standards (recombinant protein) were added to appropriate wells and incubated at 37 °C for 1 h. Biotinylated anti-IL-10 antibody (BD Biosciences) was added and plates incubated at 37 °C for 30 min. Streptavidin-horse radish peroxidase (Zymed Technologies, Pittsburgh, PA, USA) was added to each well at a dilution of 1 in 3000 in blocking buffer, and incubated at 37 °C for 20 min. Tetramethylbenzidine substrate (Invitrogen) was then added to each well and left for 2–3 min for a cyan color to develop. The reaction was stopped with 1 N H_2_SO_4_, and read at 450 nm in a plate reader.

### RNA extraction

Total RNA isolation was carried out using NucleoSpin RNA kit (Macherey-Nagel, Düren, Germany) according to the manufacturer's protocols for RNA purification from cultured cells and tissue. Briefly, samples were lysed and filtered to reduce viscosity and clear the lysate. The solution was treated with 70% ethanol to change the RNA binding conditions and the lysate loaded into a NucleoSpin RNA column and centrifuged. Membrane desalting buffer was then added, followed by DNase reaction mixture. The membrane was washed and RNA eluted from the membrane. The quantity of RNA was estimated by ultraviolet spectroscopy using a NanoDropTM spectrophotometer with NanoDrop software version 3.0.1 (Thermo Fisher Scientific, Waltham, MA, USA). RNA was extracted from cultured macrophages of three healthy controls.

### Reverse transcription and quantitative PCR

SuperScriptTM III Reverse Transcriptase kit (Invitrogen) was used as per manufacturer's instructions for first-strand complementary DNA synthesis from total RNA. Complementary DNA was diluted 1:4 in RNase-free water at the end of the reaction.

PCR reaction mix was made by mixing TaqMan Universal Master Mix II with UNG (2X), NOS2 (Hs01075529_m1) or IL12R (Hs01073447_m1) TaqMan Assay (20X) primer (GAPDH primer for control (Hs03929097_g1)), and RNase-free water. Complementary DNA was added to each well and incubated with the following steps: 2 min hold at 50 °C followed by a 10-min hold at 95 °C. Forty cycles of denature (95 °C, 15 s) and anneal/extend (60 °C, 1 min) were then completed. Quantitative PCR data were analyzed using a ViiATM 7 Real time PCR instrument, and ViiA 7 software (version 1.2, Life Technologies, Carlsbad, CA, USA). Relative quantity expression was calculated using the delta delta CT equation and normalized to the housekeeping gene, GAPDH.

### Conditioned media production

Tissue samples were mechanically dissociated and then incubated overnight in RPMI-10 (37 °C, 5% CO_2_). The tissue suspension was removed from the tube and filtered with a 70 μm cell strainer (BD Falcon) followed by a 0.45 μm filter (ReliaPrep, Munktell and Filtrak GmbH, Bärenstein, Germany) to remove all cells. Conditioned media were stored at −80 °C until use. Macrophage cultures were supplemented with conditioned media at a ratio of 1:3 with RPMI+M-CSF/GM-CSF. Cytokine content was assessed by LEGENDPLEX Multi-analyte flow assay kit (BioLegend) according to the manufacturer's instructions for IFN-γ, IL-10, TNF-α and IL-6. Tumor and NTB tissue (to prepare conditioned media) from five individual patients were studied in the experiments shown here and cultured with PBMCs from the same patient. At the end of culture, cells were analyzed for surface protein expression by flow cytometry as for *ex vivo* isolated cells.

### Statistical analysis

All statistical analyses were calculated using GraphPad Prism 6.0a (La Jolla, CA, USA). Wilcoxon test was used for matched patient samples. Mann–Whitney test was used for conditioned media comparisons. Correlations were determined using a Spearman's rank correlation coefficient. Statistical significance of cytokine concentration (measured by ELISA) was determined using a Friedman test and Dunn's multiple comparisons *post test*.

## Figures and Tables

**Table 1:** Patient characteristics

Number	11	
Age	Mean±s.d.	77.09±6.32
Gender	M:F	6:5
Tumor site	Right colon	3
	Transverse colon	3
	Left colon	3
	Rectum	2
Differentiation	Poor	1
	Moderate	9
	Mucinous	1
Stage	I	2
	IIA	3
	IIC	1
	IIIB	3
	IIIC	1
	IVA	1
Histology nodes (total)	Mean±s.d.	19.18±5.04
Percent nodes positive	Mean±s.d.	9.16±18.02
MMR	Low^a^	8
	PMS-2 negative^b^	1
	MLH-1 negative PMS-2 negative^c^	2

Abbreviations: F, female; M, male; MMR, mismatch repair; MSI-H, microsatellite instability-high.

a No loss of nuclear expression of MMR proteins: low probability of MMR proteins: low probability of MSI-H.

b Loss of nuclear expression of PMS-2 only: high probability of Lynch syndrome.

c Loss of nuclear expression of MutL homolog (MLH-1) and postmeiotic segregation (PMS-2). BRAF V600E (immunohistochemistry (IHC)): positive: excludes Lynch syndrome.
