# Supplementary figures and images for: Gut macrophage phenotype is dependent on the tumor microenvironment in colorectal cancer
Source: Clin Transl Immunology. 2016 Apr 29;5(4):e76–. doi: 10.1038/cti.2016.21 (PMC4855270; doi:10.1038/cti.2016.21)

Norton et al  
Supp Figure 1

A

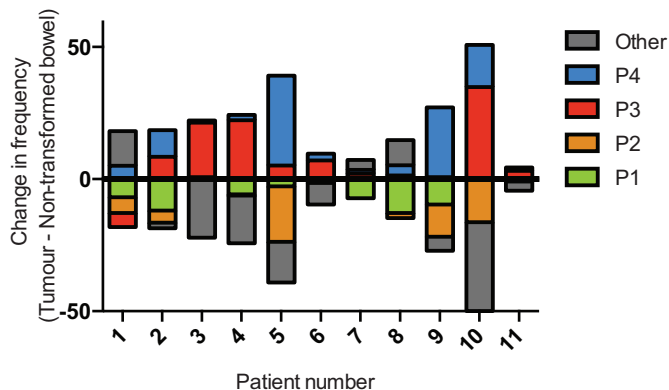

B

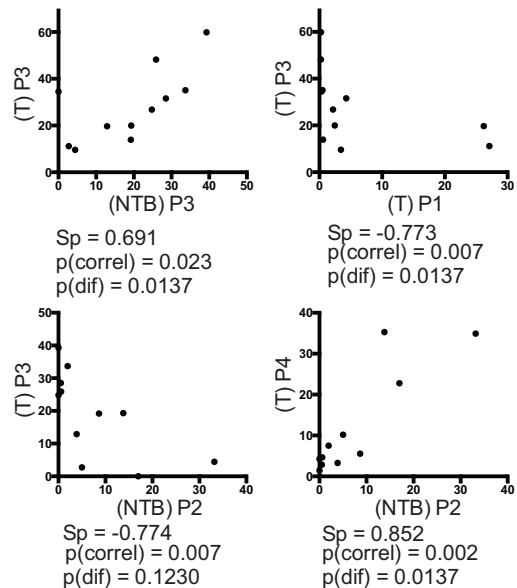

Supplement: Supplementary Figure 1 [file cti201621x1.pdf]
