# Supplementary material for: Gut macrophage phenotype is dependent on the tumor microenvironment in colorectal cancer
Source: Clin Transl Immunology. 2016 Apr 29;5(4):e76–. doi: 10.1038/cti.2016.21 (PMC4855270; doi:10.1038/cti.2016.21)
Supplement: Supplementary Figure Legend [file cti201621x2.docx]

**Supplementary Figure 1 Relative change in frequencies of macrophage populations in tumor and non-tumor bowel**

Non-tumor bowel tissue (NTB) and tumor tissue was removed by surgery from CRC patients. Macrophage cell infiltrate from NTB and tumor was quantified by flow cytometry as described in Methods. (**A**) Relative frequencies of populations P1-P4. The frequency of population in tumor tissue minus frequency of population in NTB for 11 patients is shown. (**B**)The correlation between indicated populations of macrophages P1-P4 in tumor (T) or NTB. Statistical analysis was completed using the non-parametric Spearman’s correlation coefficient. n =11.
